# Supplementary material for: The Survival Outcomes, Prognostic Factors and Adverse Events following Systemic Chemotherapy Treatment in Bone Sarcomas: A Retrospective Observational Study from the Experience of the Cancer Referral Center in Northern Thailand
Source: Cancers (Basel). 2023 Mar 26;15(7):1979. doi: 10.3390/cancers15071979 (PMC10092999; doi:10.3390/cancers15071979)
Supplement: Supplementary file 1 [file cancers-15-01979-s001.zip › cancers-2209559-supplementary.pdf]

Supplementary Table S1. The estimated 5-year DFS and 5-year OS of osteosarcoma patients by the Flexible parametric survival model.

Prognostic factors	5-year disease free survival		5-year overall survival	
	aHR (95% CI)	*p* value	aHR (95% CI)	*p* value
**Gender**				
Female	0.33 (0.16-0.72)	**0.005**	0.41 (0.20-0.87)	**0.019**
Male	(ref.)		(ref.)	
**Age group (years)**				
≥ 18 years	0.60 (0.26-1.37)	0.224	0.64 (0.51-2.14)	0.269
< 18 years	(ref.)		(ref.)	
**Resectable**	0.17 (0.07-0.40)	**<0.001**	0.21 (0.09-0.50)	**<0.001**
**Unresectable/metastasis**	(ref.)		(ref.)	
**Received neoadjuvant chemotherapy**				
TNR ≥ 90%	0.08 (0.01-0.62)	**0.016**	0.09 (0.01-0.74)	**0.024**
TNR 50-89%	2.37 (0.84-6.73)	0.105	1.59 (0.58-4.34)	0.365
TNR < 50%	0.58 (0.23-1.48)	0.257	0.71 (0.29 -1.69)	0.436
**Not received neoadjuvant chemotherapy**	(ref.)		(ref.)	
**Radiation therapy**	1.47 (0.71-3.01)	0.319	1.38 (0.66-2.85)	0.338

Abbreviations: aHR, Adjusted hazard ratio; TNR, Tumor necrosis rate (post neoadjuvant chemotherapy).

Supplementary Table S2. The estimated 5-year DFS and 5-year OS of Ewing's sarcoma patients by the Flexible parametric survival model.

**Prognostic factors**	**5-year disease free survival**		**5-year overall survival**	
	**aHR (95% CI)**	***p* value**	**aHR (95% CI)**	***p* value**
**Gender**				
Female	0.46 (0.14-1.49)	0.194	0.69 (0.19-2.46)	0.566
Male	(ref.)		(ref.)	
**Age group (years)**				
≥ 25 years	6.05 (1.46-25.10)	**0.013**	6.79 (1.33-34.72)	**0.021**
< 25 years	(ref.)		(ref.)	
**Resectable**	0.16 (0.04-0.67)	**0.012**	0.06 (0.01-0.51)	**0.010**
**Unresectable/metastasis**	(ref.)		(ref.)	
**Radiation therapy**	0.49 (0.14-1.66)	0.253	0.36 (0.08-1.62)	0.183

Abbreviations: aHR, Adjusted hazard ratio; CMT, chemotherapy.
